# Encéphalocèle occipitale et anomalies associées: colobome palpébral bilatéral, fente labio-palatine bilatérale et bride amniotique sur la jambe droite avec absence des orteils sur pieds droit et gauche aux Cliniques Universitaires du Graben Butembo 2021 (à propos d’un cas)

**DOI:** 10.11604/pamj.2022.42.251.33736

**Published:** 2022-08-03

**Authors:** Matthieu Aza Sumai, Alexandre Amini Mitamo, Joël Bosomi Yawi, Alpha Kavuyiro Muhindo, Jackson Musumba Kambale, Faida Muliwavyo Kavugho, Alexis Kahatane Kahindo, Claude Masumbuko Kasereka

**Affiliations:** 1 Département de Chirurgie, Université Catholique du Graben, Butembo, République Démocratique du Congo,; 2 Département d´Ophtalmologie, Université Catholique du Graben, Butembo, République Démocratique du Congo

**Keywords:** Encéphalocèle, colobome, fente labio-palatine, bride amniotique, cas clinique, Encephalocele, coloboma, cleft lip and palate, amniotic band, case report

## Abstract

L´encéphalocèle est une malformation due à un défaut de fermeture du tube neural associé à une hernie du tissu cérébral et/ou des méninges à travers ce défect crânien congénital. La taille d´une encéphalocèle varie de quelques centimètres à une énorme masse appelée «encéphalocèle géante». Habituellement le contenu de la malformation est composé de tissus nerveux dégénératif, des méninges et une partie kystique. Un nourrisson de 4 mois, de sexe féminin, née des parents non consanguins, nous était référée d´une structure sanitaire de la place pour prise en charge d´une masse occipitale congénitale. A l´arrivée dans notre consultation (4 mois après sa naissance), l´examen avait révélé un poids de 3500g, un périmètre crânien de 33 cm avec une fontanelle antérieure non bombée. Elle avait une énorme masse occipitale rénitente de 43 x 25 cm et d´une taille de 15 cm. une absence des deux paupières; deux fissures labiopalatines; une constriction à la jambe droite, une absence des orteils des pieds droit et gauche. Ainsi était posé le diagnostic d´une maladie des brides amniotiques avec comme composantes: encéphalocèle occipitale associée à un colobome palpébral bilatéral, une fente labio-palatine bilatérale, et des brides amniotiques sur la jambe droite, et amputation des orteils des pieds droit et gauche.

## Abstract (en)

Encephalocele is a malformation due to a defect in the closure of the neural tube causing herniation of brain tissue and/or meninges through this congenital skull defect. The size of encephalocele varies from a few centimeters to a huge mass called “giant encephalocele”. Content usually consists of degenerative neural tissue, meninges and a cystic part. We here report the case of a 4-month-old infant, born of non-consanguineous parents, who was referred with congenital occipital mass. Upon arrival at our clinic, clinical examination revealed a weight of 3500g, a head circumference of 33 cm, with a non-bulging anterior fontanelle. She presented with a huge renitent occipital mass measuring 43X25cm, with a height of 15cm was found. absence of both eyelids, bilateral lip and palate fissures; a constriction ring on the right leg, absence of toes on the right and left feet. A diagnosis of an amniotic band syndrome was made, with as components: occipital encephalocele associated with a bilateral palpebral coloboma, a bilateral cleft lip and palate, and amniotic bands on the right leg, and amputation of the toes of the right and left feet.

## Introduction

L'encéphalocèle est une anomalie rare du tube neural, survenant dans 1 naissance sur 5 000 dans le monde, pour laquelle 70% ont une localisation occipital [1]. L´encéphalocèle est une malformation due à un défaut de fermeture du tube neural faisant hernie du tissu cérébral et /ou des méninges à travers ce défect crânien congenital [2]. La taille d'une encéphalocèle varie de quelques centimètres à une énorme masse appelée «encéphalocèle géante» [3]. Habituellement le contenu de la malformation est composé de tissus nerveux dégénératif, des méninges et une partie kystique [4]. Classiquement, les encéphalocèles sont rares dans les pays développés [3] car dans ces derniers, la prévention par le conseil génétique et la prise d´acide folique en période periconceptionnelle, la précision du diagnostic anténatal, la légalisation de l´avortement thérapeutique ont fait baisser la prévalence des défect du tube neural [5]. Alors qu´elles représentent un sérieux problème de santé publique dans les pays en développement et particulièrement en Afrique subsaharienne à cause du mauvais suivi prénatal, du mariage consanguin, du bas niveau socioéconomique et du retard diagnostique [6]. Nous rapportons un cas d´encéphalocèle occipitale associée à un colobome palpébral bilatéral, une fente labio-palatine bilatérale, et des brides amniotiques sur la jambe droite, et absence des orteils des pieds droit et gauche chez un nourrisson de quatre mois. Ce rapport souligne l´importance d´une évaluation clinique minutieuse, le rôle crucial de l´imagerie cérébrale dans le diagnostic, et la possibilité d´une prise en charge multidisciplinaire (neurochirurgie réparatrice, ophtalmologique et pédiatrique) qui le plus souvent fait défaut dans le contexte local aux ressources limitées.

## Patient et observation

**Information de la patiente:** un nourrisson de 4 mois, né des parents non consanguins, nous était référé d´une structure sanitaire de la place pour pour prise en charge d´une masse occipitale congénitale. La mère, une primipare de 30 ans, n´avait rapporté aucune pathologie pendant la grossesse mais nous avait signalé la prise des médicaments au premier trimestre pour des malaises gravidiques en ambulatoire fait de: diclofénac injectable, diazépam et acide folique pendant 7 jours. Elle avait participé à 3 séances de consultation prénatale, dont les deux premières au deuxième trimestre de la grossesse et la dernière au troisième trimestre. Elle avait reçu un traitement prophylactique fait de sulfadoxine + pyrimethamine contre le paludisme, un déparasitage au mebendazol et une supplémentation en acide folique. Les échographies obstétricales prénatales n´ont pas été faites. Une césarienne a été réalisée dans un hôpital de la place dès que la grossesse était estimée à terme. Elle s´était soldée par la naissance d´un nouveau-né polymalformé de sexe féminin, pesant 2600 g, APGAR score 8/9/10. Les malformations portaient sur la tête, les yeux, la bouche et nez, ainsi que les membres inférieurs. Aucun traitement n´était tenté jusqu´à sa consultation aux cliniques Universitaires du Graben, à l´âge de 4 mois.

**Figure 1:**
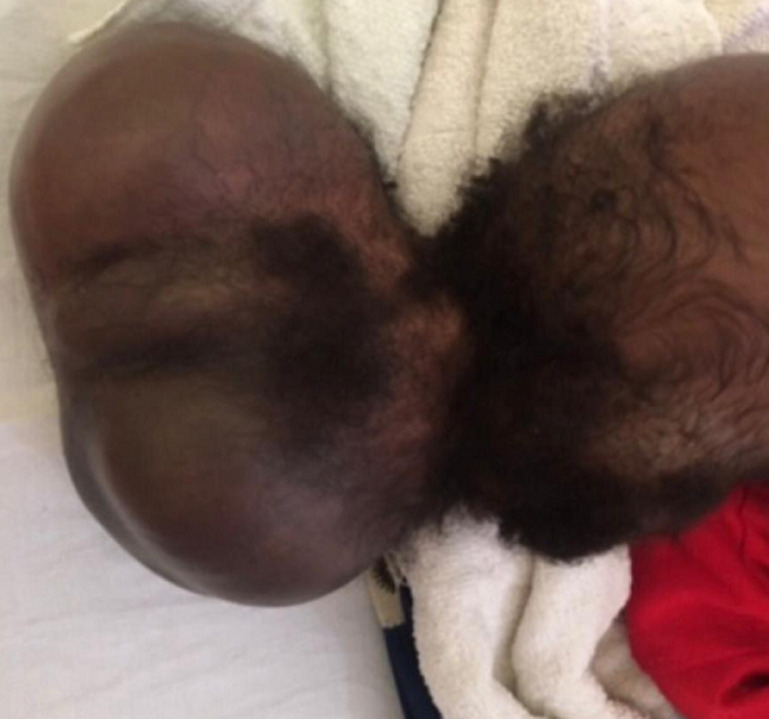
volumineuse encéphalocèle occipitale

**Figure 2:**
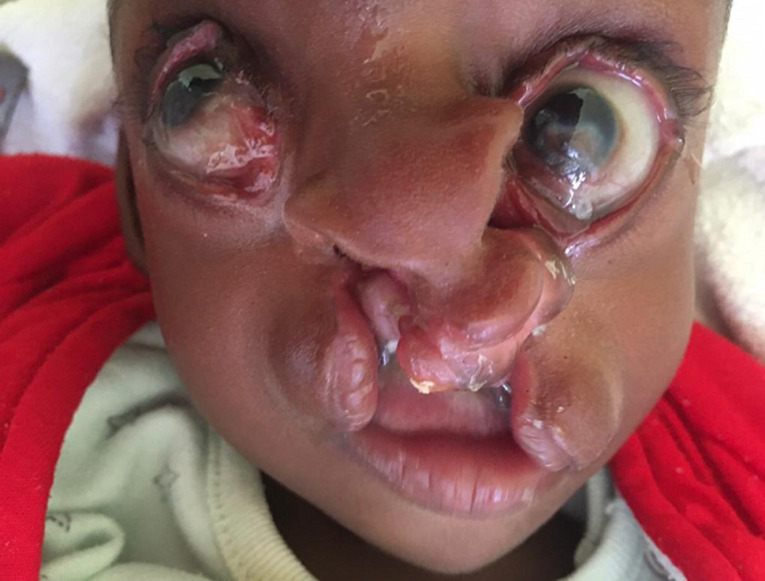
colobome palpébral bilatéral et fente labio-palatine bilatérale

**Figure 3:**
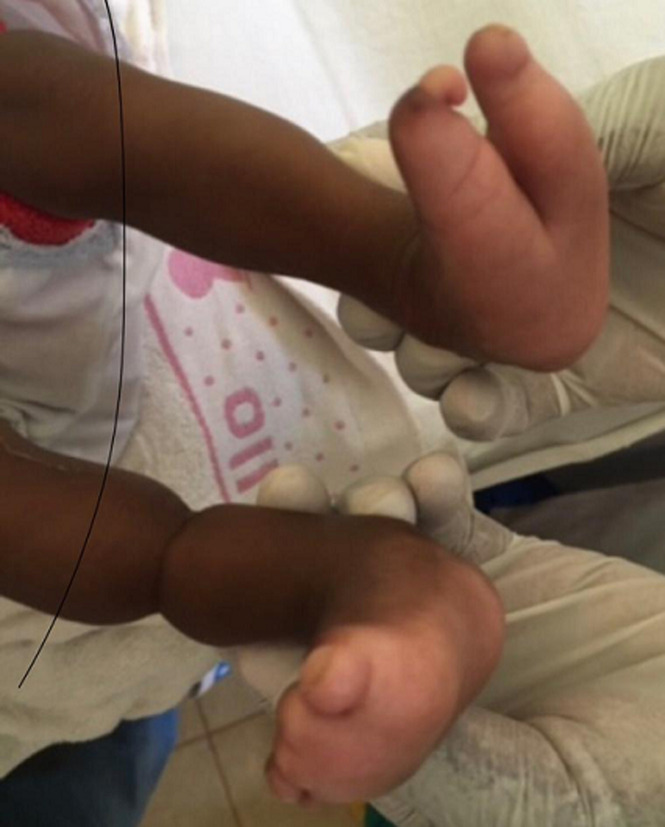
syndactylie du pied gauche et bride amniotique de la jambe droite avec agénésie des orteils

**Figure 4:**
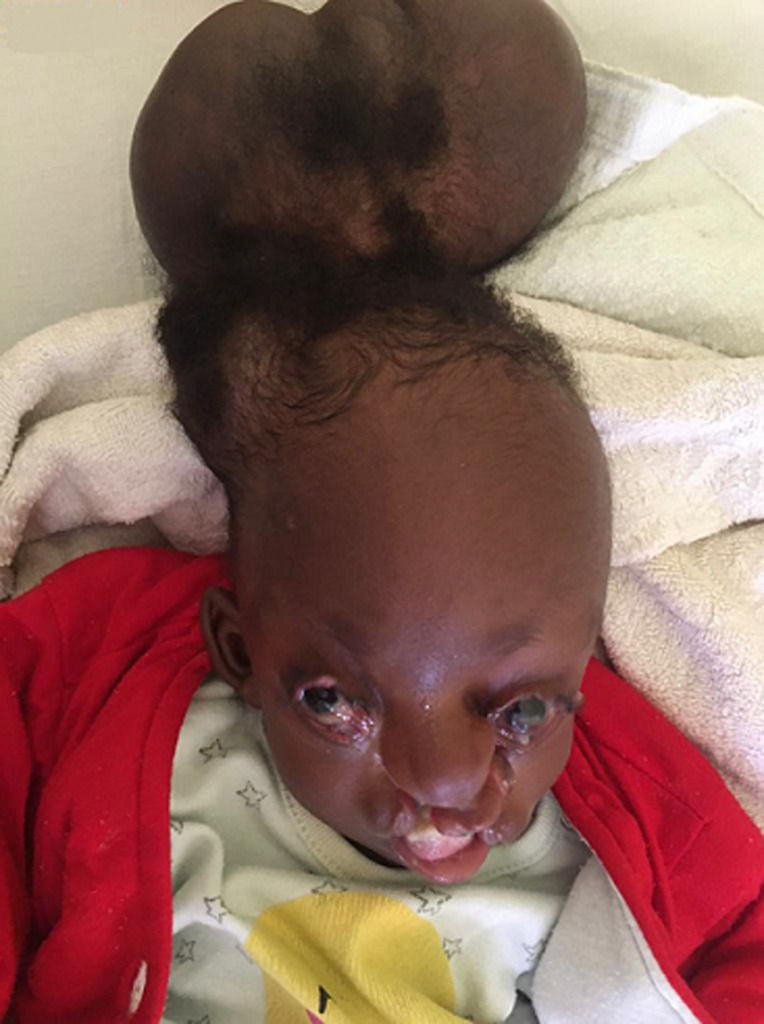
encéphalocèle occipitale, colobome palpébral, fente labio-palatine bilatérale

**Résultats cliniques:** à l´arrivée dans notre consultation (4 mois après sa naissance), l´examen clinique avait révélé un poids de 3500g, un périmètre crânien de 33 cm avec une fontanelle antérieure non bombée. Le nourrisson semblait actif. L´examen neurologique était normal. Elle avait une énorme masse occipitale rénitente de 43 x 25 cm et d´une taille de 15 cm (Figure 1). L´examen de la face avait révélé l'absence de deux paupières de chaque côté, et la présence de deux fissures labiales supérieures s'étendant jusqu'au palais (Figure 2). A ces constats crâniofaciaux s´ajoutaient des anomalies des membres pelviens ci-après: un sillon cutané de striction circonférentiel à la jambe droite, à son un tier moyen, sans lymphœdème distal ; une absence des orteils au pieds droit excepté l´hallux et une fente avant-médio pied gauche avec absence des orteils (Figure 3). Le reste de l´examen morphologique externe était normal. Ainsi été posé le diagnostic de la maladie des brides amniotiques avec comme composantes: encéphalocèle occipitale associée à un colobome palpébrale bilatéral, et brides amniotiques Patterson I à la jambe droite et Patterson IV aux deux pieds (orteils amputés) chez un nourrisson de 4 mois (Figure 4) (Tableau 1).

**Tableau 1:** classification des brides amniotiques selon Patterson (1961)

Type I	Simple sillon de constriction cutané
Type II	Sillon de constriction cutanée associé à une déformité de la partie distale du membre, avec ou sans lymphœdème
Type III	Sillon de constriction cutanée associé à une soudure des parties distales allant jusqu’à une acrosyndactylie
Type IV	Amputations intra-utérines

**Figure 5:**
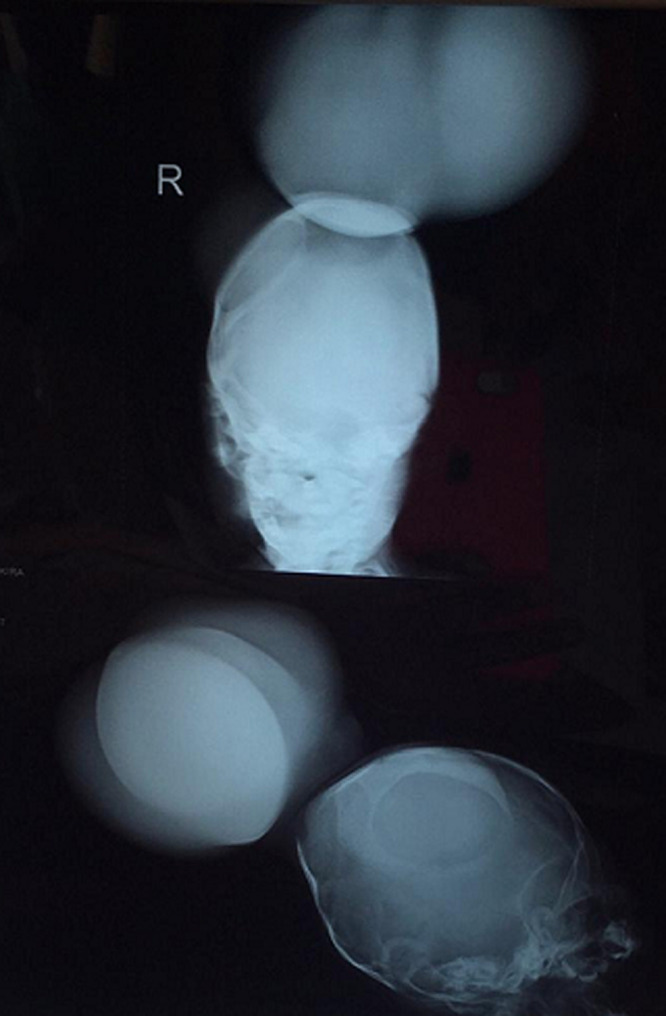
radiographie du crane incidence face et profil montrant une ouverture sur os occipital

**Démarche diagnostique:** à défaut d'un scanner cérébral, nous avons réalisé une échographie de la masse occipitale qui a montré la présence de deux masse polylobés à contenu mixte (hydrique et solide) avec prédominance solide. La radiographie du crâne incidence face et profil avait mise en évidence un défet occipital avec hernie du contenu cérébral (Figure 5). L´échographie Doppler de la jambe n´était pas réalisée étant donné que la striction était simple, sans déformation ni lymphœdème distal. Aussi, aucun bilan génétique n´était réalisé, du fait de l´absence d´un centre de biologie moléculaire dans la région.

**Intervention thérapeutique:** une conscientisation des parents était faite, portant sur les investigations ultérieures et les étapes de prise en charge du complexe malformatif de la patiente. Néanmoins, les parents n´avaient pas consenti. Ainsi, aucune intervention thérapeutique n´était réalisée chez ce patient.

**Consentement du patient:** il a été obtenu auprès des parents de la patiente.

## Discussion

Les encéphalocèles se définissent comme une hernie du tissu cérébral et/ou des méninges hors de la boîte crânienne à travers un défect osseux congénital due à un défaut de fermeture de la partie crâniale du tube neural [2]. Les encéphalocèles, les spina bifida et les anencéphalies sont communément regroupés sous le terme d´anomalies congénitales du tube neural (ACTN) [2,7]. L´étiopathogénie des encéphalocèles reste encore controversée et plusieurs théories sont avancées, telles que hyperthermie maternelle, l´acide valproïque, hypervitaminose A, la carence en vitamine B12 et en acide folique [2,7,8]. Un gène important associé à l'encéphalocèle occipitale est le CEP290 (Centrosomal Protein 290) [9]. Les encéphalocèles occipitale sont fréquemment associées à des troubles neurologiques , certains nourrissons peuvent être asymptomatiques à l'examen physique [10], mais d'autres peuvent présenter de nombreux et différents signes et symptômes tels que des retards dans l'atteinte des étapes du développement, une déficience intellectuelle, des troubles d'apprentissage, des retards de croissance, des convulsions, une déficience visuelle, un manque de coordination des mouvements volontaires (ataxie), hydrocéphalie, paraplégie spastique ou quadriplégie et microcéphalie [11]. L´incidence des encéphalocèles varie en fonction de la zone géographique et du niveau socio-économique[12,13]. Aux USA, et en Europe de l´Ouest, l´incidence des encéphalocèles est de 1 à 3 cas pour 10.000 naissances vivantes [14].

Au Canada, la prévalence de l´encéphalocèle est de 0,4 à 4 cas pour 10.000 naissances [15]. Singh *et al*. [2] avaient rapporté une prévalence globale des ACTN de 3,76 pour 10.000 naissances vivantes; alors que d´autres études menées en Asie, au Moyen-Orient et en Afrique ont rapporté une prévalence qui varie entre 23 et 61 cas pour 10000 naissances vivantes [2,16,17]. En effet, plusieurs études en Afrique subsaharienne confirment la fréquence élevée des encéphalocèles à cause du bas niveau socio-économique, de la consanguinité, du mauvais suivi de la grossesse et du jeune âge de la mère [2,18]. Les efforts consentis en matière de prévention de cette affection par la supplémentation en acide folique dans la période periconceptionnelle et l´amélioration de la couverture sanitaire nationale sont des éléments qui expliqueraient la baisse de cette malformation au Niger [17]. Ndoma *et al*. [19] en Centrafrique avaient rapporté respectivement 7 cas/an et 4,44 cas/an. Radouani *et al*. au Maroc [18], avaient rapporté 68 cas d´anomalies de fermeture du tube neural dont un seul cas d´encéphalocèle en 4 ans. L´association des encéphalocèles avec d´autres anomalies telles que l´hydrocéphalie, la microcéphalie, le retard psychomoteur et intellectuel, le syndrome de Chiari de type II, la maladie de Dandy-Walker est fréquente [20]. L´échographie anténatale dans des mains entrainées est l´examen de choix dans le dépistage anténatal des malformations cérébrales. Elle permet de détecter le défect crânien avec parfois une hernie du cerveau. Elle montre une masse sur la ligne médiane du crâne plus souvent dans la zone occipitale que frontale [21].

Les mesures préventives adoptées dans plusieurs pays développés, telles que la prise de l´acide folique dans la période periconceptionnelle, le diagnostic anténatal par l´imagerie (échographie ou IRM), la législation de l´interruption thérapeutique de grossesse sont des éléments qui concourent à réduire la prévalence des ACTN [2,22]. Dans notre cas, la mère avait pris l´acide folique pendant la période periconceptionnelle. Au Niger, des efforts de sensibilisation ont été effectués dans le cadre de la prévention des ACTN [16]. En Ethiopie, la majorité des mères, soit 85,3% (151/177), n'avaient jamais reçu une supplémentation en acide folique. Moins de 1% (2/177) des mères avait commencé à prendre une supplémentation en acide folique avant la grossesse [17]. De nombreux auteurs recommandent fortement la mise en œuvre des stratégies nationales de prévention afin de réduire la prévalence des ACTN par la prise de l´acide folique en période periconceptionnelle [6,13,23]. La réparation chirurgicale des encéphalocèles peut être effectuée en toute sécurité lorsque les conditions techniques sont réunies. L´objectif est d´assurer une fermeture étanchée, physiologique et cosmétique. L´approche chirurgicale varie et le piège commun est la fermeture insuffisante de la dure-mère entraînant une fuite de liquide céphalorachidien (LCR) en post-opératoire ou la formation d´un pseudoméningocèle [1,4]. Le traitement chirurgical précoce avant l'installation de dysmorphies cranio-faciales importantes donne des résultats cosmétiques satisfaisants [6,1,4].

## Conclusion

L'encéphalocèle occipitale est la forme la plus courante de l'encéphalocèle et se manifeste par une tuméfaction congénitale de différentes tailles sur l'os occipital sur la ligne médiane. Sa découverte chez un patient nécessite la recherche des malformations associées, qui dans certains cas constitue le complexe maladie des brides amniotiques. Le diagnostic repose principalement sur l'utilisation de techniques de l'imagerie. La chirurgie est la meilleure option pour le traitement et le moment approprié se situe entre la naissance et 4 mois. La prise en charge globale débute par la sensibilisation de la population pour une consultation rapide et surtout un affermissement des stratégies de prévention par la supplémentation en acide folique dans la période periconceptionnelle, un renforcement de notre plateau technique et une approche multidisciplinaire pour ce qui est de la correction finale.

## References

[1] Stéphanie A, Black JA, Galvez MA, Schwartz RAJ (2014). Images en anesthésiologie: voies respiratoires (Prise en charge chez un nourrisson atteint d'une encéphalocèle occipitale géante). Anesthésiologie.

[2] Singh K, Johnson WMS, Archana R, Kumar A (2016). The prevalence and pattern of neural tube defects and other major congenital malformations of nervous system detected at birth in Barbados. J Anat Soc India.

[3] Ghritlaharey RK (2018). Un bref examen de l'encéphalocèle occipitale géante. Journal des neurosciences en milieu rurals' entraîner.

[4] Archer NP, Langlois Ph, Suarez L, Brender J, Shanmugam R (2007). Association of paternalage with prevalence of selected birth defects. Birth Defects Res A Clin Mol Teratol.

[5] Raja RA, Qureshi AA, Memon AR, Ali H, Dev V (2008). Pattern of encephaloceles: a case series. JAyub Med Coll Abbottabad.

[6] Kabré A, Zabsonre DS, Sanou A, Bako Y (2015). The cephaloceles: a clinical, epidemiological and therapeutic study of 50 cases. Neurochirurgie.

[7] Padmanabhan R (2006). Etiology, pathogenesis and prevention of neural tube defects. Congenit Anom.

[8] Oucheng N, Lauwers F, Gollogly J, Draper L, Joly B, Roux F-E (2010). Frontoethmoidal meningoencephalocele: appraisal of 200 operated cases: Clinical article. J Neurosurg Pediatr.

[9] (2022). MalaCards: the human disease database, Occipital Encephalocele. MalaCards: the human disease database.

[10] Agarwal A, Chandak AV, Kakani A, Reddy S (2010). A giant occipital encephalocele. APSP J Case Rep.

[11] Rehman L, Farooq G, Bukhari I (2018). Neurosurgical Interventions for Occipital Encephalocele. Asian J Neurosurg.

[12] Rifi L, Barkat A, El Khamlichi A, Boulaadas M, El Ouahabi A (2015). Neurosurgical management of anterior meningo-encephaloceles about 60 cases. Pan Afr Med J.

[13] Sanoussi S, Chaibou M, Bawa M, Kelani A, Rabiou M (2009). Encéphalocèle occipitale: aspects épidémiologiques, cliniques et thérapeutiques: à propos de 161 cas opérés en 9 ans à l´hôpital national de Niamey. Afr J Neurol Sci.

[14] Hervey-Jumper SL, Cohen-Gadol AA, Maher CO (2011). Neurosurgical management of congenital malformations of the brain. Neuroimaging Clin N Am.

[15] Lo BW, Kulkarni AV, Rutka JT, Jea A, Drake JM, Lamberti-Pasculli M (2008). Clinical predictors of developmental outcome in patients with cephaloceles. J Neurosurg Pediatrics.

[16] Bhandari S, Sayami JT, K C RR, Banjara MR (2015). Prevalence of congenital defects including selected neural tube defects in Nepal: results from a health survey. BMC Pediatr.

[17] Sorri G, Mesfin E (2015). Patterns of neural tube defects at two teaching hospitals in Addis Ababa, Ethiopia a three years retrospective study. Ethiop Med J.

[18] Radouani MA, Chahid N, Benmiloud L, Elammari L, Lahlou K, Barkat A (2015). Epidémiologie et facteurs de risque des anomalies de fermeture du tube neural: données marocaines. Pan Afr Med J.

[19] Ndoma VN, Gaudeuille A, Nganguene J, Nghario Jl, Issa-Mapoukaetude A (2016). Des malformations du tube neural: spina bifida et encephalocele dans le service de chirurgie pédiatrique de Bangui. Rev CAMES Sci Santé.

[20] Warf BC (2011). Hydrocephalus associated with neural tube defects: characteristics, management, and outcome in sub-Saharan Africa. Childs Nerv Syst ChNS Off J Int Soc Pediatr Neurosurg.

[21] El Mhabrech H, Ben Mansour S, Dakkem M, Zrig A, Ben Hmida H, Hafsa C (2016). Diagnostic anténatal de l´encéphalocèle. J Neuroradiol.

[22] Copp AJ, Stanier P, Greene NDE (2013). Neural tube defects: recent advances, unsolved questions, and controversies. Lancet Neurol.

[23] Bergman JEH, Otten E, Verheij J, de Walle HEK (2016). Folic acid supplementation influences the distribution of neural tube defect subtypes: a registry-based study. Reprod Toxicol.

